# Regulation of cell cycle drivers by Cullin-RING ubiquitin ligases

**DOI:** 10.1038/s12276-020-00508-4

**Published:** 2020-10-02

**Authors:** Sang-Min Jang, Christophe E. Redon, Bhushan L. Thakur, Meriam K. Bahta, Mirit I. Aladjem

**Affiliations:** Developmental Therapeutics Branch, Center for Cancer Research, National Cancer Institute, NIH, Bethesda, MD 20892-4255 USA

**Keywords:** Drug development, Targeted therapies, Origin firing, Chromatin remodelling, Post-translational modifications

## Abstract

The last decade has revealed new roles for Cullin-RING ubiquitin ligases (CRLs) in a myriad of cellular processes, including cell cycle progression. In addition to CRL1, also named SCF (SKP1-Cullin 1-F box protein), which has been known for decades as an important factor in the regulation of the cell cycle, it is now evident that all eight CRL family members are involved in the intricate cellular pathways driving cell cycle progression. In this review, we summarize the structure of CRLs and their functions in driving the cell cycle. We focus on how CRLs target key proteins for degradation or otherwise alter their functions to control the progression over the various cell cycle phases leading to cell division. We also summarize how CRLs and the anaphase-promoting complex/cyclosome (APC/C) ligase complex closely cooperate to govern efficient cell cycle progression.

## Cell biology: Cell cycle mediators offer therapeutic targets

A large group of protein complexes involved in regulating cell division offers an array of promising new drug targets for treating cancer and neurodegeneration. Mirit Aladjem, Sang-Min Jang and colleagues from the US National Cancer Institute in Bethesda, USA, review the structure and function of cullin-RING ubiquitin ligases (CRLs), a “super-family” of multi-subunit enzymes that serve as critical checkpoints on the cell cycle and DNA replication. CRLs promote progression through the cell cycle by targeting other key proteins for degradation or modification. They also work with other protein complexes to ensure genome integrity in the face of DNA damage. Because CRL components are often mutated or otherwise dysregulated in tumors and other diseased cells, small-molecule inhibitors and modulators of CRL activity could have therapeutic benefit for patients.

## Introduction

Eukaryotic cell proliferation is determined by a highly organized series of steps that make up the mitotic cell cycle, consisting of DNA synthesis (S phase) and mitosis (M phase) separated by gap phases (G1 and G2). Progression through these four phases of the cell cycle is precisely modulated by proteins whose activity and stability can be regulated through posttranslational modifications such as ubiquitination^1,2^. Ubiquitination is a process whereby ubiquitin molecules are attached to protein lysine residues to regulate protein localization, recycling or degradation. Ubiquitin conjugation is catalyzed by an enzymatic cascade initiated with the activation of a ubiquitin molecule by the E1 ubiquitin-activating enzyme. Next, ubiquitin is transferred to the E2 enzyme through a thioester-linked E2-ubiquitin intermediate. Finally, an E3 enzyme identifies and recruits the targeted substrate protein, interacts with the E2-ubiquitin intermediate, and catalyzes the transfer of ubiquitin to the targeted protein. Ubiquitination can be reversed by deubiquitinating enzymes, which can cleave ubiquitin from a modified lysine. Polyubiquitinated protein substrates can then be subjected to hydrolysis by the 26S proteasome^2^, but ubiquitination can serve a purpose other than marking proteins for degradation (see examples below). Moreover, proteins can be degraded without being ubiquitinated^1^.

**Fig. 1:**
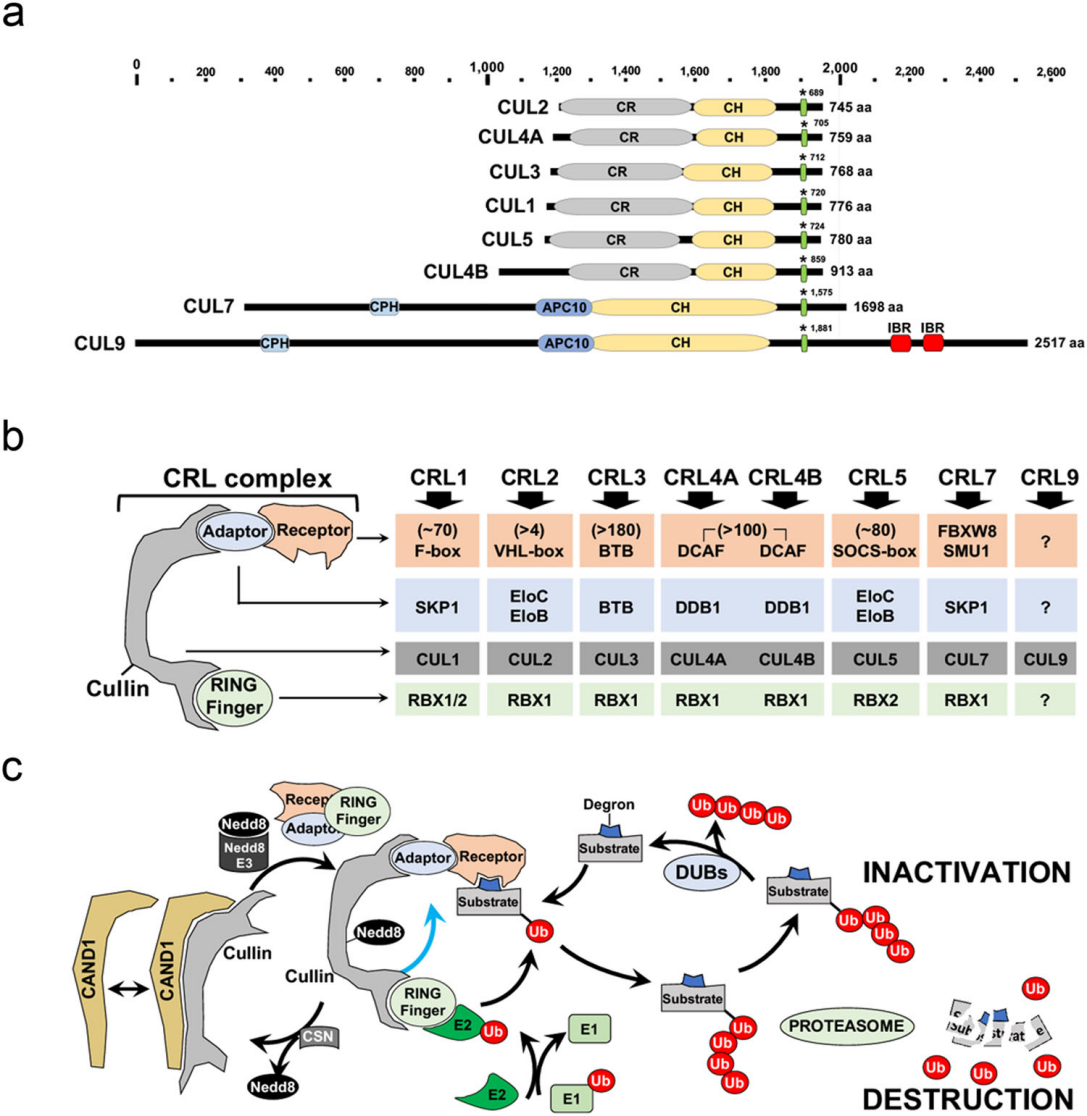
**a** Schematic representation of human cullin structural domains. Cullins are displayed by size with the smallest cullin (CUL2) on top and aligned based on their neddylation site (asterisk). CRL domain information was retrieved from InterPro (https://www.ebi.ac.uk/interpro/). CH Cullin homology domain; CR cullin repeats. CR are very flexible and may account for CRL conformation change after the activation of target substrates; CPH, CUL7, PARC, and HERC2-containing domain; APC10, a domain homologous to a sequence element termed the DOC domain and found in proteins that mediate ubiquitination reactions. **b** Each CRL protein complex is formed from a scaffold protein or Cullin (Cullin 1, 2, 3, 4A, 4B, 5, 7 or 9), a RING finger protein (RBX1 or RBX2), an adaptor protein (SKP1 for CRL1 and CRL7 or EloC/EloB for CRL2 and CRL5, BTB for CRL3, DDB1 for CRL4s and FBXW8 or SMU1 for CRL7) and a receptor-substrate recognition protein (F-box family for CRL1 and CRL7, VHL family member for CRL2, BTB for CRL3, DCAF family for CRL4s and SOCS family for CRL5). Approximate number of known CRL receptors for each indicated CRL. **c** CRL complex dynamics. Deneddylated and inactive free cullins can bind to CAND1. Cullin neddylation, which is catalyzed by a NEDD8-activating enzyme and ligase and ubiquitin-conjugating enzyme, allows cullins to interact with other CRLs when released from CAND1. Neddylation is believed to induce conformational changes in CRLs (blue arrow), which leads to their interaction with the E2-ubiquitin complex and substrate ubiquitination. Ubiquitination often leads to proteolytic degradation or substrate inactivation. Cullin neddylation is reversed by the COP9/CSN signalosome, which triggers CRL disassembly.

Mammalian cells express eight classes of CRL complexes, each containing four core components: one of eight cullin isoforms serving as a common backbone scaffold protein; a RING-containing E2-conjugation enzyme (RBX1 or RBX2) that binds to the C-terminus of cullin; an adaptor protein that binds to the N-terminus of cullin; and a substrate receptor that recognizes the ubiquitination target^3^ (Fig. 1). Cullins 1–5 and 8 share similar structural and domain features and range in size from 745 to 913 amino acids. Cullin 7 and Cullin 9 (also known as PARC) are larger than (1698 and 2517 amino acids, respectively*)* and distinct from the other cullins. In addition to the cullin homology domain found in all cullins, both CUL7 and CUL9 contain a p53-binding domain or CPH (CUL7, PARC and HERC2-containing) domain and an APC10/DOC domain similar to that found in the APC/C ubiquitin ligase complex (Fig. 1a). This structure contributes to the possibility that CRL7 and CRL9 may have functions redundant to those of APC/C. CRL activity is regulated by various mechanisms, including the sequestration of cullins by CAND1, the conjugation of the ubiquitin-like protein NEDD8 at lysine residues located in the C-termini of cullins, and the degradation of CRL components.

The adaptor subunits of CRL serve as links between substrate receptors and cullins. Some adaptors are shared among CRLs. For example, SKP1 can be shared by both the CRL1/SCF and the CRL7 complexes, Elongin B and C are the adaptor proteins for CRL2 and CRL5, and DDB1 is an adaptor protein for CRL4A and CRL4B^3^. CRL3 interacts with several BTB (Bric-a-brac, Tramtrack, Broad-complex) domain-containing proteins that exert dual functions as adaptors and substrate receptors^3^. The BTB domain in SLX4 has a similar dual function, modulating SLX4 SUMOylation activity^4^. Cells contain a multitude of substrate receptors that interact with the various CRLs; these receptors are critical for the specificity of a given CRL for its target proteins. To date, at least 70 F-box protein receptors have been identified for CRL1, a handful of VHL domain-containing proteins for CRL2: approximately 180 BTB proteins for CRL3; more than 100 DDB1- and CUL4-associated factor (DCAF) proteins for CRL4A and CRL4B; 40 SOCS proteins for CRL5; and two proteins, FBXW8 and SMU1, for CRL7^3^ (Fig. 1). It is reasonable to think that combinations of cullins, adaptors, and substrate receptors can generate hundreds of different CRL complexes that may, in turn, target thousands of substrates. Many distinct CRLs play critical roles in countless cellular processes ranging from DNA repair to cell proliferation, chromatin remodeling, DNA replication, DNA transcription, cell differentiation, metabolism, and cell migration^3^.

In this review, we describe how CRLs promote cell cycle progression by targeting key proteins for degradation or modification of function. We also summarize how CRLs and the APC/C cooperate closely to govern efficient cell cycle progression.

## CRLs target key proteins for cell cycle progression

### CRLs target CDK inhibitors

Progression through the cell cycle is mediated by protein kinases known as cyclin-dependent kinases (CDKs). CDKs are either activated by cyclins and CDC25A/B phosphatases or inhibited by CDK inhibitors (CKIs) of the INK4 and CIP/KIP families. The INK4 inhibitors encompass four known members (p15, p16, p18, and p19) that inhibit CDK4 and CDK6. CIP/KIP inhibitors consist of three known members, p21, p27, and p57, which target CDK1, CDK2, CDK4 and CDK6. CKIs are strong regulators of G1, S and G2 phase transitions; overexpression or deficiencies of these proteins lead to cell cycle arrest or to cell cycle checkpoint failures, respectively. CKIs are regulated by various mechanisms, including transcription, mRNA stability, epigenetic silencing, and ubiquitin-dependent and ubiquitin-independent protein degradation.

**Fig. 2:**
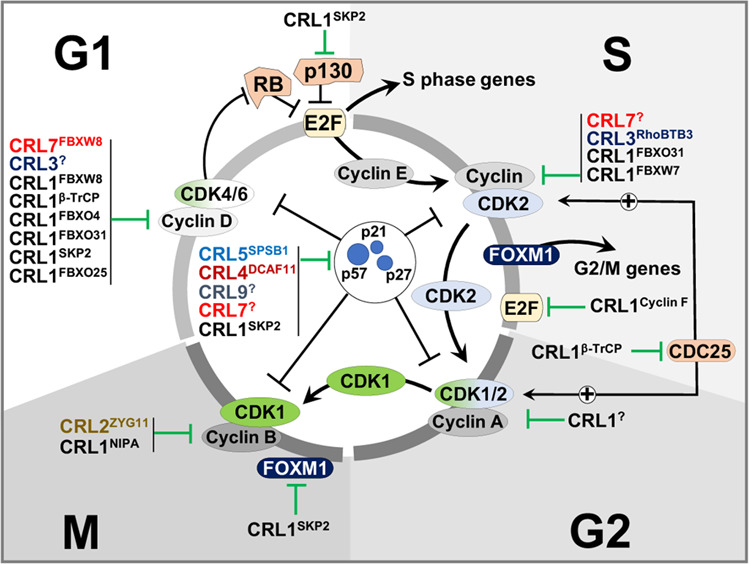
Depiction of key proteins targeted by CRLs during cell cycle progression. CDK complexes drive cell cycle progression. CDK levels remain stable throughout the cell cycle phases, whereas fluctuations in the levels of CDK activators (CDC25A/B and cyclins) and inhibitors (p21, p27, and p57) cause changes in CDK activities. Thus, CRL-mediated destruction of CDK-regulating proteins allows the disassembly and assembly of different cyclin-CDK complexes in the cell cycle phases (transfer from the CDK2-Cyclin E complex in S phase to CDK2-Cyclin A in G2 phase and from CDK1-Cyclin in G2 phase to CDK1-Cyclin B in M phase). CRLs also control cell cycle progression by the degradation of pocket proteins such as RB and p130, in turn promoting the activation of E2F transcription factors and the expression of genes needed for subsequent phases of the cell cycle, including EMI1, Cyclin A, and Cyclin E. In late S, E2Fs are targeted for destruction by CRL1, promoting cell cycle progression to G2 while preventing premature S-phase entry. Substrates targeted by CRLs are symbolized with green inhibition symbols. CRLs with superscripts denote the specific CRL-receptor complexes involved is a particular pathway. The “+” sign denotes CDK activation by CDC24A/B (by dephosphorylation).

E3 ubiquitin ligases play essential roles in the degradation and regulation of CKIs during cell cycle progression. During the G2/M phase, the targeting of CKIs for degradation is primarily mediated by the APC/C E3 ubiquitin ligase complex. At other phases of the cell cycle, CRL complexes also act to ubiquitinate and target CKIs for degradation (Fig. 2). The CRL1 complexes have an especially broad spectrum of CKI targets during interphase. At the end of the G1 phase, the ubiquitination and degradation of p21, p27, and p57 by the substrate receptor SKP2 associated with CRL1 (hereafter noted CRL1^SKP2^) promotes the G1/S transition and DNA replication by derepressing CDK2 kinase^5–7^. Accordingly, SKP2-knockout mice exhibit high p27 levels^8^.

Other CRLs are known to target CKIs. CRL4^CDT2^ targets p21 specifically in S phase when it is bound to PCNA, thus boosting S-phase progression^9^. The CRL4^DCAF11^ complex promotes S phase progression by targeting p21, thus also derepressing CDK2. In *Drosophila* and *Xenopus laevis*, CRL4 also regulates p27 degradation^10^. In human cells, the degradation of cytoplasmic p21 by CRL2^LRR1^ may not have a role in cell cycle progression but may play a role in actin-based cell movement^11^. CUL5^SPSB1^ interacts and promotes the ubiquitin-mediated proteasomal degradation of p21 in ovarian cancer cells. CUL5^SPSB1^ also regulates p21 mRNA levels, which suggests that SPSB1 might be involved in regulating p21 transcription (e.g., through transcription factors or other chromatin components)^12^. Both CRL7 and CRL9 may also have a direct impact on CDK activity since the silencing of CUL7 and CUL9 leads to dysregulated p21/p27 and p21 protein expression in human cancer cells, respectively. In summary, the degradation of p21, p27, and p57 by CRLs can modulate CDK activities at all phases of the cell cycle.

### CRLs target CDK activators

#### Cyclins

Cyclins drive the events of the cell cycle by binding and activating CDKs. The five major families of cyclins (Cyclin A, B, C, D, and E) are defined based on the fluctuation of their protein levels during the cell cycle. Cyclins C and D govern the exit from quiescence and the progression through the G1 phase, while E-type cyclins are master switches for entry into the S phase. Finally, Cyclin A controls DNA replication and progression through the G2 phase, and B-type Cyclins dictate entry into mitosis and chromosome segregation. Most CRLs play substantial roles in cell cycle progression by targeting cyclins. CRL redundancy for cyclin degradation may be linked to cell line/tissue specificities and/or may enable the rapid and accurate adjustment of cyclin levels.

Early hints at the roles of CRLs in cyclin level regulation were discovered nearly three decades ago in yeast when mutations in *GRR1*, an F-box protein component of CRL1, resulted in the stabilization of the yeast G1 cyclins Cln1, Cln2, and Cln3^13^. It was later discovered that two F-box proteins, GRR1 and CDC4, both components of the CRL1 complex, bind and redundantly target Cln3 for degradation^14^. GRR1 and CDC4 bind to two different epitopes of the Cln3 C-terminus in a CDK-phospho-dependent manner. While the two F-box proteins can also bind Cln1 and Cln2, only Cln2 is targeted by GRR1 for degradation. F-box specificity for G1 cyclins can be explained partly by the cellular localization of cyclins (i.e., nuclear *vs*. cytoplasmic). Cells derived from mice with the CRL1 substrate receptor β-TrCP1 knocked out showed mitotic defects and abnormal amplification of the centrosome accompanied by the stabilization of Cyclin A, Cyclin B, and EMI1^15^.

Cyclin D (D1, D2, and D3) has similar functions in mammalian and yeast cells. Cyclin D activates CDK4 and CDK6. Their levels increase in G1 and accumulate until reaching the G1/S-phase boundary. Upon entry into S phase, Cyclin D loss of stability is critical for DNA replication^16^. In G1, Cyclin D1 binds PCNA and directly prevents DNA replication, while in late G1, the Cyclin D/CDK4 pair (and the Cyclin E-CDK2 pair later in S phase) phosphorylates and inactivates the pocket proteins retinoblastoma (RB), retinoblastoma-like 1 (p107), and retinoblastoma-like 2 (p130). The phosphorylation of these pocket proteins in turn releases them from inhibitory interactions with the E2F1, E2F2, E2F4 and E2F3A transcription factors (E2Fs)^17,18^ (Fig. 2). In turn, activated E2Fs promote the gene expression needed for progression through S and G2, including the expression of Cyclins E and A^19^. E2Fs are targeted for degradation in late S phase by the CRL1^Cyclin F^ complex^20^. Their destruction is thought to prevent premature S-phase exit. The activities of E2Fs are also governed by the targeted degradation of RB and p130 by CRL2^ZYG11^ and CRL1^SKP2 ^^21^.

Several CRL1 complexes, including CRL1^SKP2^, CRL1^FBXO4^, CRL1^FBXW8^, CRL1^FBXO25^, and CRL1^FBXO31^, target Cyclin D^7,22,23^. Upon entry into S phase, Cyclin D is phosphorylated on threonine 286 (Thr286) by glycogen synthase kinase 3β (GSK3β), which triggers interactions between Cyclin D and CRL1 substrate receptors^24^. Thr286-phosphorylated Cyclin D is exported into the cytoplasm, where it is then able to interact with CRL complexes and undergo ubiquitin-mediated degradation^25^. In human prostate and human hepatoma cells, Cyclin D1 is targeted by the CRL1 complex, CRL1^β-TrCP^, in response to berberine and STG28, a PPARγ-inactive troglitazone derivative^26^. Interestingly, no association between Cyclin D1 and SKP2, FBXW7, FBXO4, or FBXW8 was observed after STG28 treatment, suggesting that the targeting of specific substrate recognition subunits for Cyclin D1 destruction may be determined by a specific cellular stress. Finally, two atypical CRL1 complexes, PCF4/FBXO4 and PCF7/FBXW7, containing PARK2 but not SKP1 or RBX1 were shown to be involved in both Cyclin D and Cyclin E ubiquitination, respectively^27^. Other CRL families share overlapping functions with CRL1 for Cyclin D degradation, either directly or indirectly. For example, CUL4B (CRL4) silencing leads to a decrease in Cyclin D1 levels and G1 arrest^28^, while CRL1^β-TrCP^ promotes the degradation of DYRK1A, a nuclear protein kinase that facilitates Cyclin D phosphorylation-induced degradation^29^.

Members of the E-type cyclin family (Cyclin E1 and Cyclin E2) accumulate during the G1/S transition and are completely degraded by the end of the S phase. Cyclin E binds to and activates CDK2 to control S phase entry and progression^30,31^. Several CRL families govern Cyclin E levels. CRL1^FBXW7^ and CRL3^RhoBTB3^ may target CDK2-bound phosphorylated Cyclin E and free Cyclin E^32^. CRL4 also plays a role in Cyclin E destruction via the interaction and polyubiquitination of Cyclin E, as CUL4B silencing in *Drosophila* leads to increased Cyclin E levels^33^. Degradation of Cyclin E by CRLs may promote the switch from one CDK activity to another (i.e., freeing CDK2 from Cyclin E will enhance its assembly with Cyclin A to regulate the progression to the next phase of the cell cycle) (Fig. 2).

The two subtypes of Cyclin A, Cyclin A1 and Cyclin A2, have functions that were thought to be restricted to meiosis and mitosis^30^. However, Cyclin A1 may also have a function in some mitotic cells^34^. Cyclin A expression is initiated upon entry into the S phase and peaks during the G2/M phases to induce mitosis^30^. Cyclin A binds and activates CDK1 and CDK2, thus regulating separate functions in the S and G2 phases^30^. CRL1^SKP2^ is critical for Cyclin A destruction^35^. The degradation of Cyclin A by CRL1 frees CDK1 from Cyclin A/CDK1 and allows it to interact with Cyclin B to form a complex that is critical for G2/M progression.

Cyclin G2 is highly expressed in terminally differentiated tissue^36^. Nevertheless, growing evidence suggests that Cyclin G2 is involved in mitosis^11^ and may contribute to cell cycle progression, with Cyclin G2 overexpression leading to cell cycle arrest in the G1 phase^37^. CRL1 may be involved in Cyclin G2 destruction since SKP1 and SKP2, two components of CRL1, bind Cyclin G2, and SKP2 and Cyclin G2 levels show a negative correlation^38^.

#### CDC25 phosphatases

CDC25 dual-specificity phosphatases (CDC25A, CDC25B, and CDC25C) remove inhibitory phosphate groups on CDKs. Through this dephosphorylation, CDC25 phosphatases activate CDK-cyclin complexes and promote cell cycle progression from the G1 to M phase. CDC25 phosphatases are unstable proteins whose cellular levels are regulated by alternating synthesis and ubiquitin-mediated proteolysis. While the APC/C ubiquitin ligase complex degrades the greatest percentage of CDC25 during mitotic exit and in early G1, CRL1^β-TrCP^ controls the protein levels of CDC25 in the S and G2 phases^39^. Accordingly, β-TrCP1/2 silencing causes CDC25 accumulation and hyperactive CDK2 activity^40^. The interactions between APC/C-CDC25A and CRL1-CDC25A require different CDC25A recognition motifs^39,41^. Action through CDC25A exemplifies how CRLs can have opposite effects on cell cycle progression, with CRL1 complexes acting as both positive (degradation of CKIs) and negative regulators (degradation of CDC25).

## CRLs regulate DNA replication

During DNA replication, dozens of proteins and enzymes act cooperatively to rapidly and accurately duplicate the genetic information of a cell. CRLs regulate this process through the degradation of key proteins at critical steps. DNA replication involves three major steps: initiation, elongation, and termination. In eukaryotes, the initiation of replication begins during late mitosis and early G1 phase with the loading of the origin recognition complex (ORC, a highly conserved six-subunit origin recognition complex, ORC1/6) to potential replication origins^42^. Replication origin licensing is then finalized by the subsequent recruitment of minichromosome maintenance 2–7 (MCM2–7) helicases, which is facilitated by the interaction of the licensing factors CDC6 and CDT1 with the chromatin-bound ORC^42^. Entry into the S phase is associated with MCM2-7 activation by cyclin-dependent kinases, a step that facilitates the recruitment of additional components and allows the replicative helicase CMG (CDC45/MCM2–7/GINS) to undertake DNA replication. To ensure cell survival and to avoid genomic instability, no chromosomal DNA loci can be replicated more than once per cell cycle. To prevent such DNA re-replication, proteins critical for origin licensing and/or activation are degraded by CRLs after the initiation of DNA replication.

### CDC6 and CDT1

CRLs promote the ubiquitination of proteins involved in the initiation of replication, such as CDC6 and CDT1 and are necessary to regulate origin licensing and to avert DNA re-replication. In mammalian cells, CDC6 is targeted by CRL4^CDT2^ once cells enter the S phase and by CRL1^Cyclin F^ in G2 and early mitosis^43,44^. The involvement of CRLs in the ubiquitination and degradation of CDT1 has been studied extensively. In most eukaryotes, the critical licensing factor CDT1 is sequestered or targeted for degradation after the initiation of DNA replication utilizing two separate pathways to prevent new origin licensing and DNA re-replication. It is sequestered by the protein regulator Geminin and targeted for degradation by CRLs. Timely ubiquitination and degradation of CDT1 are carried out by several redundant pathways utilizing at least two CRLs: CRL1 and CRL4. CDT1 ubiquitination-mediated degradation is promoted by CRL4^CDT2^ at the G1/S transition and during ongoing replication, while the CRL1^SKP2^ and CRL1^FBXO31^ complexes are involved in CDT1 degradation in the S/G2 phases^45–48^. CRL4 can target CDT1 for degradation in the G1 phase upon DNA damage^49^. CUL4A and CUL4B appear to have overlapping functions in CRL4-induced CDT1 degradation, as the expression of both proteins needs to be silenced simultaneously to protect CDT1 from degradation^47^. Chromatin loading of CRL4 requires RepID (DCAF14/PHIP), a protein shown to be important for the initiation of a subset of replication origins. CRL4 is recruited to chromatin by RepID during the G1 phase of the cell cycle, whereas in the S phase, it is recruited by PCNA^47^. In the absence of RepID, cells rely on CRL1^SKP2^ for CDT1 degradation^47^.

Substrate receptors for CRL1 and CRL4 complexes (SKP2 and CDT2, respectively) recognize two different regions located in the first 100 amino acid sequence of CDT1. Phosphorylation of CDT1 at a conserved N-terminal threonine residue (Thr-29) is required for CDT1 degradation by the CRL1^SKP2^ complex^49^. It is most likely that this CDT1 phosphorylation is carried out by Cyclin A-CDK2 in early S phase and by Cyclin A-CDK1 in late S and G2^50^. Efficient degradation of CDT1 by CRL4 requires the interaction of CDT2 with proliferating cell nuclear antigen (PCNA) through a consensus PCNA-interaction protein (PIP) box, a motif in several PCNA‐interacting proteins^45^. CDT1 also binds to PCNA through its own PIP box. By directly interacting with both CDT2 and CDT1, PCNA is believed to function as a molecular platform that brings the CRL4 complex into proximity with its substrate CDT1 and thus facilitates the ubiquitination of CDT1 by CRL4 in S phase. PCNA may also function to similarly promote CRL4-mediated destruction of other proteins^51^.

### MCM2-7 replicative helicase

All six subunits of the MCM2–7 heterohexamer have been reported to be ubiquitinated in vivo^52^. MCM2-7 ubiquitination may alter MCM2-7 protein-protein interactions and/or activation. In addition, MCM2-7 ubiquitination plays a role in replication termination. In yeasts, MCM3 degradation is dependent on the CRL1 complex associated with the F-box receptor GRR1. MCM3 degradation is regulated by CDK, with CDK-induced phosphorylation of MCM3 leading to MCM exclusion from the nucleus, where CRL1 is predominantly located. CDK-induced exclusion of MCMs from the nucleus also serves as a backup system for preventing DNA re-replication. In mammalian cells, MCM3 is ubiquitinated by the CRL3^KEAP1^ complex^53^. CUL3^KEAP1^-induced MCM3 ubiquitination does not target MCM3 for degradation, nor does KEAP1 silencing or overexpression change MCM3 levels or cellular compartment distribution. Since MCM4 and MCMBP contain a KEAP1-binding motif, it is reasonable to think that CRL3^KEAP1^ may ubiquitinate additional MCM proteins^54^. MCMBP may replace MCM2 in MCM helicases^55^ and/or might function by unloading the MCM complex from chromatin at the end of the S phase, possibilities that strongly suggest a role for CRL3^KEAP1^ in different aspects of the replication process.

Studies in yeast and *Xenopus* have shown that MCM7 is ubiquitinated, a process that leads to the disassembly of the replication machinery during DNA replication termination^56^. The ubiquitination of MCM7 during replication termination requires CRL1^DIA2^ in budding yeast^57^, while MCM7 degradation in metazoans is initiated by CRL2^LRR1 58^. In the absence of CRL2^LRR1^, unloading of the replicative CMG helicase from chromatin is inhibited, and other components of the replisome, including DNA Pol ε, are retained on DNA^58^.

### Other target proteins involved in DNA replication

CRLs target a myriad of other proteins involved in DNA replication whose aberrant levels may affect S-phase progression and cell proliferation. A non-exhaustive list of these proteins is listed in Table 1. For example, MCM10, an MCM2-7-interacting protein that acts as a scaffold to promote DNA replication and protect against replication stress^59^, is targeted by the CRL4^VprBP^ complex in response to stress^60^. DNA topoisomerases I and II, enzymes that resolve topological stress during DNA replication^61^^,^ are regulated by CRL3, CRL4, and CRL2/VHL. PCNA, TopBP1, subunit p12 of DNA polymerase Pol δ, and DNA ligase I are targeted for degradation by CRL4, CRL2, CRL4^CDT2^ and CRL4^DCAF7 ^^62^, while CUL-3 and CRL1^SLMB^ play redundant roles in the rhythmic ubiquitination of TIMELESS^63^.

**Table 1:** Non-exhaustive list of CRLs, receptors and their substrates involved in cell cycle progression.

CRLs	Substrates	Receptors	Substrate roles	Reference
CRL1	p21	SKP2	Cyclin-dependent kinase (CDK) inhibitor. Degradation regulates progression into various phases of the cell cycle	^7^
CRL1	p27	SKP2	CDK inhibitor. Degradation regulates progression into various phases of the cell cycle	^8^
CRL1	p57	SKP2	CDK inhibitor. Degradation regulates progression into various phases of the cell cycle	^6^
CRL1	Cyclin A	SKP2	Cyclin A regulates CDK2 (S phase) and CDK1 (G2/M) activities	^35^
CRL1	Cyclin B1	NIPA	Cyclin B regulates CDK1 activity in G2/M phases	^98^
CRL1	Cyclin D1	FBXO4 FBXW8 FBXO25 β-TrCP FBXO31 SKP2	Cyclin D regulates CDK1 activity in G2/M phases	^7,22–24,26^
CRL1	Cyclin D2, D3	FBXL2	Cyclin D regulates CDK1 activity in G2/M phases	^22,73^
CRL1	Cyclin E	FBXW7	Cyclin E regulates CDK2 activity, Important for G1/S and S phase progression	^106^
CRL1	Cyclin F	β-TrCP	CRL1 substrate recognition. Regulates cell cycle progression in S and G2/M	^77^
CRL1	CDC25A	β-TrCP	Cyclin-dependent kinase activator. Controls entry into various phases of the cell cycle	^39^
CRL1	p130	SKP2	Derepresses E2Fs, allowing progression through S and G2	^107^
CRL1	E2F1, E2F2, E2F3A, E2F7	Cyclin F	Activates transcription of genes crucial for S and G2/M progressions	^20^
CRL1	CDC6	Cyclin F	Initiation of DNA synthesis. Its destruction inhibits DNA rereplication	^43^
CRL1	CDT1	FBXO31 SKP2	Initiation of DNA synthesis. Its destruction inhibits DNA rereplication	^47,49^
CRL1	MCM7	DIA2	Ubiquitination leads to disassembly of MCM helicase	^57^
CRL1	TIMELESS	SLMB	Part of the replisome.	^63^
CRL1	JMJD2A	FBXL4	Degradation during S and G2	^108^
CRL1	SET8	β-TrCP	Degradation in G1 phase	^70^
CRL1	Histone MacroH2A.1	SKP2	Chromatin component. Ubiquitination leads to chromatin remodeling	^66^
CRL1	SLBP	Cyclin F	SLBP degradation in G2 to inhibit histone accumulation	^68^
CRL1	Securin	?	Chromosome separation in Mitosis.	^100^
CRL1	EMI	β-TrCP	Inhibits APC/C. Its destruction allows progression through mitosis	^15^
CRL1	Aurora A, B	FBXL2, 7	Regulate Aurora kinases abundance for normal mitosis	^94^
CRL1	CP110	Cyclin F	Required for normal centrosome duplication	^76^
CRL1	FOXM1	FBXO31	Transcription factor active in G2 for transcription of genes crucial for progression through G2/M	^75^
CRL1	WEE1	β-TrCP	Inhibits CDK1 in mitosis. Its degradation allows progression through mitosis	^109^
CRL1	SAK/PLK4	β-TrCP	Controls centriole duplication. Destruction allows normal mitosis progression	^78^
CRL1	MYC	FBXW7	Accumulation of cells in S-phase and G2/M phase	^110^
CRL1	JunB	FBXW7	Destruction inhibits premature sister chromatid separation	^79^
CRL1	DYRK1A	β-TrCP	G1 to S progression	^29^
CRL2	p21	LRR1	CDK inhibitor. Degradation regulates progression into various phases of the cell cycle	^11^
CRL2	Cyclin B1	ZYG11A/B	Cyclin B regulates CDK1 activity in G2/M phases	^71^
CRL2	RB	ZYG11BL	Derepresses E2Fs, allowing progression through S and G2	^21^
CRL2	MCM7	LRR1 (Xenopus)	Ubiquitination leads to disassembly of MCM helicase	^58^
CRL2	TopBP1	Ad12 E4orf6	Involved in the control of DNA replication	^62^
CRL2	SLBP	FEM1	SLBP degradation in G2 to inhibit histone accumulation	^111^
CRL2	Histone H2B	BAF250	Chromatin component. Ubiquitination leads to chromatin remodeling	^112^
CRL2	TOP2α	VHL	Alter DNA topology. Progression through mitosis	^113^
CRL3	Cyclin E	RhoBTB3	Cyclin E regulates CDK2 activity, Important for G1/S and S phase progression	^32^
CRL3	Cyclin D1	?	Cyclin D regulates CDK1 activity in G2/M phases	^114^
CRL3	MCM3	KEAP1	Ubiquitination leads to disassembly of MCM helicase	^53^
CRL3	MCM4 and MCMBP	?	Ubiquitination leads to disassembly of MCM helicase	^54^
CRL3	TIMELESS	?	Part of the replisome	^63^
CRL3	TOP1	?	Alters DNA topology. Progression through S phase	^115^
CRL3	Aurora A	KLHL18	Ubiquitination mediates mitosis entry	^116^
CRL3	Aurora B	KLHL21	Ubiquitination ensures recruitment of the chromosomal passenger complex	^117^
CRL3	PLK1	KLHL22	Crucial for G2/M transition. Ubiquitination release PLK1 from kinetochores	^118^
CRL3	P60/katanin	KLHDC5	Ubiquitination controls microtubule levels necessary for normal mitosis	^81^
CRL4	p21	DCAF11 CDT2	CDK inhibitor. Degradation regulates progression into various phases of the cell cycle	^9^
CRL4	P27	?	CDK inhibitor. Degradation regulates progression into various phases of the cell cycle	^33^
CRL4	Cyclin E	?	Cyclin E regulates CDK2 activity, Important for G1/S and S phase progression	^33^
CRL4	CDC6	CDT2	Initiation of DNA synthesis. Its destruction inhibits DNA rereplication	^44^
CRL4	CDT1	CDT2	Initiation of DNA synthesis. Its destruction inhibits DNA rereplication	^47,49^
CRL4	MCM10	VrpBP	Stress-induced MCM10 destruction and G2/M arrest	^60^
CRL4	PCNA	?	PCNA degradation induced by EGFR inhibition	^119^
CRL4	p12	CDT2	Destruction modify the DNA polymerase δ complex during S phase progression	^120^
CRL4	Ligase I	DCAF7	LIG1 degradation after inhibition of proliferation	^121^
CRL4	TOP1	?	Alters DNA topology. Progression through S phase	^122^
CRL4	CENP-A	RBBP7	Ubiquitination promotes CENP-A association to chromatin required for normal mitosis	^83^
CRL4	Histone H2A	DDB2	Chromatin component. Ubiquitination leads to chromatin remodeling	^65^
CRL4	SLBP	WDR23	Histone biogenesis	^68^
CRL4	SUPT16H	?	Ubiquitination targets FACT to DNA replication	^64^
CRL4	SET8	CDT2	Histone methyltransferase. Degradation in S phase/limit histone expression	^69^
CRL4	MMSET	CDT2	Histone methyltransferase. Degradation in S phase. Maintenance of Pre-RC complexes on chromatin	^123^
CRL4	WIPI2/ATG18B	?	Mitotic inhibition of autophagy	^84^
CRL4	BUB3	RepID/ RBBP7	Ubiquitination allows metaphase to anaphase transition	^103^
CRL5	p21	SPSB1	CDK inhibitor. Degradation regulates progression into various phases of the cell cycle	^12^
CRL5	DDA3	ASB7	Degradation controls microtubule polymerization. Required for normal mitosis progression	^124^
CRL7	p21 and p27	?	CDK inhibitor. Degradation regulates progression into various phases of the cell cycle	^125^
CRL7	Histone H2B	SMU1	Chromatin remodeling. Promote sister chromatid cohesion during mitosis	^88^
CRL7	MRFAP1	FBXW8	Promotes anaphase to telophase transition	^126^
CRL7	Cyclin D1	?	Regulates CDK1 activity in G2/M phases	^127^
CRL9	Survivin	?	Polyploidy in Cul9-depleted mice	^87^
CRL9	p21		CDK inhibitor. Degradation regulates progression into various phases of the cell cycle	^128^

Chromatin composition and modifications directly impact cell cycle progression. FACT, a heterodimeric protein complex composed of SSRP1 and SPT16, can either assemble or partially disassemble nucleosomes and affect DNA repair, transcription, and DNA replication. RTT101, the yeast homolog of human CUL4, ubiquitinates SPT16, and RTT101 deletion leads to reduced association of the replicative helicase MCM with FACT on replication origins^64^. Thus, FACT ubiquitination may promote the loading of MCMs to replication origins. Chromatin remodeling and histone biogenesis are critical for DNA replication and progression through S phase. Histones H2A, H2B, and macro H2A.1 are substrates for CRL1, CRL4 and CRL2/CRL7, respectively^65,66^. CRL4^WDR23^ catalyzes the nonproteolytic polyubiquitination and activation of SLBP (stem-loop binding protein) to ensure histone supply during DNA replication^67^. The same SLBP protein is ultimately degraded during G2 by CRL1^Cyclin F^ and CRL2^FEM1^ in vertebrates and lower eukaryotes, respectively, to inhibit re-entry into the S phase^68^.

The levels of several histone modifiers, including SET8 (H4 methylation modifier), MMSET (H3 and H4 methylase) and JMJD2A (H4 demethylase), are dynamically regulated by CRLs to promote cell cycle progression^69^. For example, the CRL4-mediated proteolytic degradation of SET8 is critical for cell cycle progression from the S phase to the G2 phase^69^. SET8 deregulation may induce re-replication through a defect in H4K20Me1 signaling at replication origins. SET8 stability is controlled by CRL4 and CRL1, with SET8 proteolysis promoted in the S and G1 phases by CRL4 and CRL1, respectively^70^.

## CRL substrate receptors determine the specificity of G2/M phase progression

The Cyclin B-CDK1 pair is part of the M phase-promoting complex that regulates progression during M phase through the phosphorylation of many substrates necessary for accurate cell division. Both the APC/C and CRL complexes cooperate to achieve Cyclin B degradation. Cyclin B can be categorized into three subtypes (Cyclin B1, Cyclin B2 and Cyclin B3). Cyclin B expression is at its peak during the G2 to M transition and is degraded during the metaphase-to-anaphase transition^30^. Cytoplasmic Cyclin B is translocated to the nucleus immediately before mitosis, where it binds and activates CDK1. The yeast Cyclin B ortholog, Cig2, is targeted by the CRL1 complex during the G2 and M phases, whereas APC/C completes Cig2 degradation during late mitosis and G1. An F-box protein homologous to the human FBXW7 protein was shown to be the CRL1 substrate receptor critical for Cig2 destruction. In mammalian cells, Cyclin B1 is the substrate of CRL1^NIPA^ in interphase only, allowing Cyclin B1 to accumulate in G2/M. Inhibition of CRL1^NIPA^ in G2/M is accomplished by NIPA phosphorylation by Cyclin B/CDK1. Thus, Cyclin B seems to contribute to its own abundance in mitosis. Cyclin B1 is also targeted by the CRL2^ZYG11A/B^ complex^71^, which is critical for Cyclin B1 degradation when APC/C is inactivated, an outcome that emphasizes cross talk between CRLs and APC/C.

**Fig. 3:**
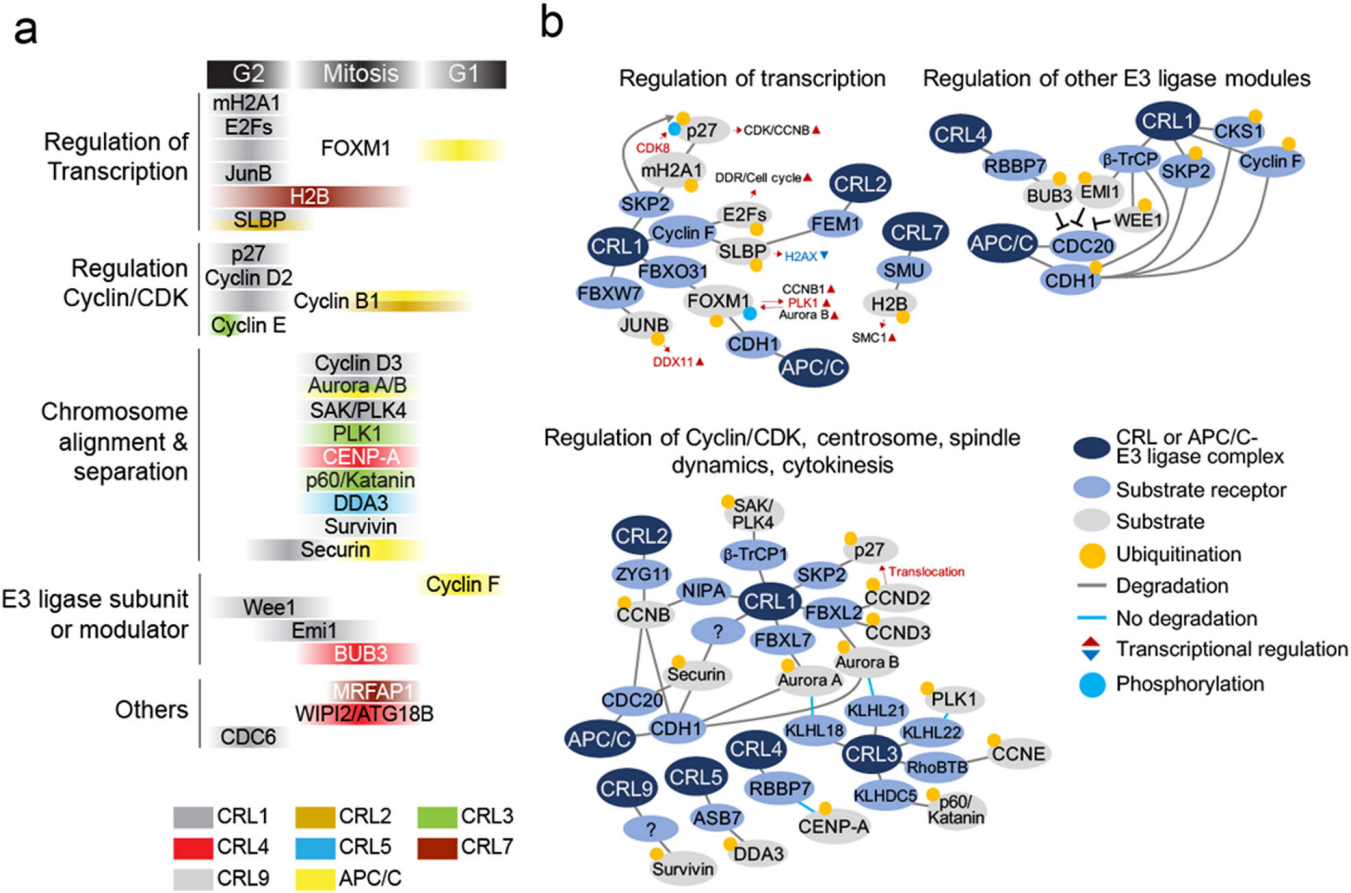
CRLs and cell cycle progression through G2/M. **a** Diagram depicting how CRLs promote timely substrate ubiquitination during G2/M. Substrates are categorized into five groups (transcription, CDK activities, chromosomes, modulators of other E3 ligases and others) and color coded to match their CRLs. **b** Schematic summary of CRLs, substrate receptors and substrates involved in the regulation of cell cycle progression through G2/M. Substrates are categorized into three groups (regulation of transcription, regulation of CDKs and mitotic factors and regulation of other E3 ligases). CRL complexes, substrate receptors, phosphorylation and ubiquitination are color coded as indicated.

In addition to Cyclin B, many other key proteins are also targeted for degradation by CRLs to promote G2/M progression. Specific substrate receptors of CRL complexes play critical roles in this process (Fig. 3). In mice, depletion of the CRL1-associated substrate receptor SKP2 leads to the accumulation of p27, resulting in cell polyploidies and centrosome overduplication. A normal phenotype is restored in SKP2 and p27 double-knockout mice, supporting the idea that CRL1^SKP2^ regulates G2/M progression by regulating p27 levels^72^. The SKP2-macroH2A1-CDK8 axis controls p27 protein expression in breast cancer cells: CRL1^SKP2^ targets macroH2A1 degradation, which in turn leads to increased CDK8 expression and CDK8-induced p27 proteolysis^66^.

In transformed lung epithelial cells, the ectopic expression of the CRL1 substrate receptor FBXL2 or knockdown of Cyclin D3 have overlapping phenotypes, including G2/M arrest, tetraploidy and the appearance of supernumerary centrosomes. The speculation that FBXL2 and Cyclin D3 belong to a common pathway was confirmed with the finding that FBXL2 binds and promotes Cyclin D3 ubiquitin-induced proteolysis^73^.

Another CRL1 substrate receptor, FBXO31, acts as a transcription factor regulator of the G2/M transition and M phase progression and is dynamically regulated in the cell cycle, with levels peaking from the late G2 to the early G1 phase^74^. FBXO31 knockdown results in mitotic arrest with increased lagging chromosomes and anaphase bridges^75^. Investigations into the mechanism of action of FBXO31 revealed that CRL1^FBXO31^ targets FOXM1 for destruction at the G2/M boundary, altering, in turn, the expression of its mitotic factor targets Cyclin B1, Polo-like kinase 1 (PLK1), and Aurora B^75^.

β-TrCP1 regulates the G2/M transition through its effect on Cyclin F. Cyclin F (FBXO1) levels oscillate throughout the cell cycle with increasing levels during S phase, peaking during G2, and finally diminishing during mitosis and G1. Depletion of Cyclin F induces centrosomal and mitotic abnormalities such as multi, bipolar- and asymmetric spindles and lagging chromosomes^76^. CRL1^β-TrCP^ binds and degrades Cyclin F in a casein kinase IIa phosphorylation-dependent manner during the G2/M transition^77^. Mice deficient for β-TrCP1 display abnormal mitosis progression that includes lengthened mitosis, centrosome overduplication, misaligned chromosomes, and multipolar metaphase spindles^78^. A central role for CRL1^β-TrCP^ in mitosis was also demonstrated in *Drosophila*. Slimb, the fly ortholog of mammalian β-TrCP, targets the destruction of a member of the polo-like kinase family (SAK/PLK4) critical for centriole formation during mitosis^78^. Accordingly, depletion of Slimb leads to centrosome amplification and mitotic abnormalities.

CRL1^FBXW7^ is essential for normal mitosis, and FBXW7 downregulation results in cells arresting in both S and G2/M phases. The role of CRL1^FBXW7^ in cell cycle progression through mitosis involves the JunB-GSK3-DDX11 axis. DDX11 is a helicase involved in chromatid cohesion, and its transcription is regulated by the transcription factor JunB, is targeted for degradation by CRL1^FBXW7^ in G2/M in a GSK3-phospho-dependent manner. Consequently, the accrued JunB levels observed in *FBXW7*^−/−^ cells lead to DDX11 accumulation and premature sister chromatid separation before anaphase^79^.

Progression through mitosis is also regulated by the CRL3 complex. During mitosis, the spindle assembly checkpoint (SAC) maintains genome stability by delaying cell division until accurate chromosome segregation is certain. This regulation is achieved through SAC activation by PLK1. Once all kinetochores are stably attached to the microtubule spindle apparatus, CUL3, together with its substrate receptor KLHL22, binds and ubiquitinates PLK1, leading to its dissociation from kinetochores and allowing SAC to be silenced and chromosomes to segregate^80^. CRL3 also controls normal mitotic progression through another mechanism involving the substrate receptor KLHDC5 (KLHL42). By targeting p60/Katanin, which functions to sever microtubules, CRL3^KLHDC5^ facilitates the maintenance of katanin at the physiological level to regulate microtubule biogenesis level (too little or too much katanin results in a buildup of microtubules or prevents microtubule formation, respectively)^81^.

CRL4 regulates mitosis through its effect on H3 variant centromere protein‐A (CENP‐A). CENP‐A replaces some of the canonical histone H3.1 variants at the inner region of centromeres and is required for the normal assembly of the kinetochore^82^. CENP‐A loss results in inaccurate chromosome segregation. CRL4^RBBP7^ promotes the loading of newly synthesized CENP‐A at centromeres during the G1 phase, and silencing of CRL4 components leads to prolonged mitotic progression, similar to the results of knocking out CENP‐A^83^.

CRL4 may also regulate mitosis through the inhibition of autophagy. Autophagy is thought to be inhibited in a spatial and time-specific manner during mitosis, possibly to protect untimely chromosome degradation and midbody ring digestion during cytokinesis. CRL4 plays a role in the mitotic inhibition of autophagy by binding to and mediating the polyubiquitination and proteasomal degradation of WIPI2, a protein facilitating the nucleation and expansion of phagophore membranes^84^.

CRLs also regulate microtubule polymerization, a critical step in the progression through mitosis. CRL5^ASB7^ targets the microtubule-associated protein DDA3 for polyubiquitination and proteasomal degradation. Optimum levels of DDA3 are critical for normal mitosis since DDA3 regulates the dynamics of the mitotic spindle, thus allowing normal chromosome alignment in metaphase. CRL7 may also have a crucial role during G2/M phase, as CUL7 depletion results in altered microtubule dynamics, prometaphase arrest, tetraploidy, and mitotic cell death^85^. Both CRL7 and CRL9 act during mitosis progression to target survivin for destruction. Survivin controls multiple steps of mitosis by recruiting the chromosomal passenger complex to mitotic chromosomes and regulating microtubule dynamics^86^. CUL9 promotes the ubiquitination and degradation of survivin and protects cells from microtubule damage. CUL7 depletion decreases survivin levels, and survivin overexpression attenuates the defects caused by CUL7 depletion. Finally, mitosis and microtubule defects caused by CUL7 depletion can be attenuated by CUL9 depletion^87^.

The CRL7^SMU1^ E3 ligase complex mediates H2B monoubiquitination. While H2B ubiquitination has been implicated in a wide range of cellular processes, such as the DNA damage response, cell differentiation and transcription, CRL7^SMU1^-induced H2B ubiquitination was reported to promote sister chromatid cohesion during mitosis by regulating SMC1 expression^88^. Another CRL7 complex, CRL7^FBXW8^, plays a critical role in the anaphase-telophase transition by degrading the Mof4 family associated protein 1 (MRFAP1). MRFAP1 levels vary during the cell cycle, being maximal in metaphase, completely disappearing in anaphase, and reappearing in telophase^89^.

## Cross talk between CRLs and APC/C ligases

**Fig. 4:**
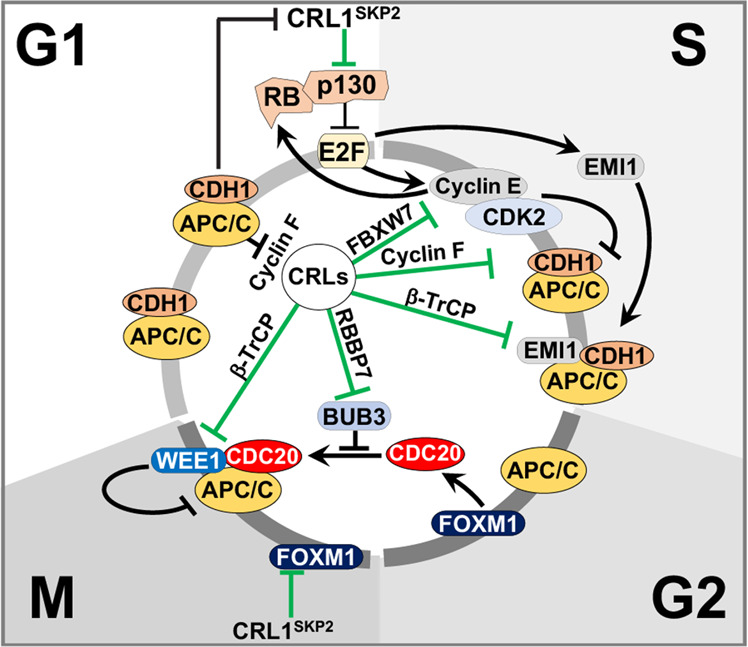
Examples of cross talk between CRLs and APC/C complex ligases for efficient cell cycle progression. CRL1^SKP2^ activity is suppressed by APC/C^CDH1^ in G1, but during the G1/S transition, Cyclin E/CDK2 inactivates APC/C^CDH1^, leading to the accumulation of SKP2. CRL1^SKP2^ and Cyclin-CDK activation of E2Fs promotes EMI1 expression, leading to APC/C inhibition throughout the S phase. The capacity of CRL1^FBXW7^ to target Cyclin E for destruction can affect APC/C activity. APC/C and CRL1^Cyclin F^ form a reciprocal feedback loop controlling cell cycle progression, with Cyclin F and CDH1 antagonizing each other. Cyclin F is targeted for ubiquitination and degradation by APC/C^CDH1^ in the G1 phase, while CDH1 itself is a substrate of CRL1^Cyclin F^ in the S phase. BUB3 prevents premature chromosome segregation by blocking APC/C from associating with its coactivator CDC20. Prior to entry into metaphase, CRL4^RBBP7^ targets BUB3 for degradation, allowing APC/C activation. WEE1, a negative regulator of APC/C^CDC20^, is targeted for degradation by CRL1^β-TrCP^ in a Cyclin B1/CDK1-dependent manner. Substrates targeted by CRLs are symbolized with green inhibition symbols (see text for details).

For proper cell cycle progression, the activities of CRLs and APC/C are coordinated, with cross talk or cooperation occurring between the two ubiquitin ligase complexes (Fig. 4). In contrast to CRLs, APC/C activity is restricted to the G2/M and G1 phases. APC/C temporal activation is controlled by its association with coactivators CDC20 (in G2/M) and CDH1 (in G1), by interactions with APC/C inhibitors, and by the phosphorylation of the APC/C core. CRL1^SKP2^ activity can be controlled by APC/C since SKP2 is a target of APC/C^CDH1^ in the G1 phase^5^. Cyclin E-CDK2 inactivates APC/C^CDH1^ during the G1/S transition, leading to the accumulation of SKP2^90^. APC/C activity is also regulated through a Cyclin E/CDH1 axis. Phosphorylation of CDH1 by Cyclin E-CDK2 governs the dissociation of CDH1 from the APC core subunit^91^. Thus, the capacity of CRL1^FBXW7^ to target Cyclin E for destruction can affect CDH1 phosphorylation status and APC/C activity.

CRL and cyclin-CDK activation of E2Fs promotes EMI1 expression, an inhibitor of APC/C, leading to APC/C inhibition throughout the S phase^92^. As cells transit through G2, EMI1 is phosphorylated by Cyclin B-CDK1, which allows to be recognized and destroyed by CRL1^β-TrCP1^. Upon EMI1 destruction, APC/C becomes active in mitosis^15^. The mitotic kinases Aurora A/B, required for the proper progression of mitosis by regulating bipolar mitotic spindle formation and chromosome segregation, are targeted by the APC/C^CDH1^, CRL^FBXL7^, CRL^FBXL2^, CRL3^KLHL18^ and CRL3^KLHL9^ complexes^93–97^. Tight control of Cyclin B1 levels is achieved by the APC/C complex in mitosis and by the CRL1^NIPA^ complex during interphase^98,99^. The cellular abundance of securin is dictated by the APC/C during metaphase and mitotic exit and by CRL1 during interphase^100^. WEE1, a negative regulator of APC/C^CDC20^, controls the timing of entry into mitosis. Cyclin B1/CDK1-phosphorylated WEE1 is targeted for degradation by CRL1^β-TrCP^, leading to Cyclin B1/CDK1-dependent APC/C activation^101^. APC/C and CRL1^Cyclin F^ form a reciprocal feedback loop controlling cell cycle progression. Cyclin F is targeted for ubiquitination and degradation by APC/C^CDH1^ in the G1 phase, while CDH1 is itself a substrate of CRL1^Cyclin F^ or CRL1^β-TrCP^ in the S phase^102^. Consequently, CRL1^Cyclin F^ and APC/C^CDH1^ antagonize each other, with CRL1 inhibiting APC/C activity in the S phase.

Cross talk between CRL4 and APC/C also occurs during metaphase (Fig. 4). The spindle assembly checkpoint (SAC) complex, which includes the mediator BUB3, plays a crucial role as a surveillance network preventing premature chromosome segregation by blocking APC/C from associating with its coactivator CDC20. Prior to entry into metaphase, RepID recruits CRL4^RepID^ to chromatin. During metaphase, chromatin-bound CRL4 dissociates from RepID and binds another substrate receptor, RBBP7. In turn, CRL4^RBBP7^ targets BUB3 for degradation, releasing SAC and allowing mitotic exit by activating APC/C^103^.

## Conclusions and perspectives

In this review, we summarize the ways in which CRLs play integral roles in the highly ordered progression of the cell cycle. The understanding of the relationship between the cell cycle and some CRL families is relatively new (i.e., CRL7 and CRL9), and overall, many questions regarding the roles of CRLs in cell cycle progression remain. Future studies are expected to disclose new CRL components, substrates new posttranslational modifications, which alter the function of these proteins. Evidence of cross talk between CRLs and other ubiquitin ligase pathway components will likely be revealed.

Ultimately, the main goal of studying the advanced CRL cellular machinery controlling the cell cycle is to develop efficient drugs that will specifically target cell cycle progression. The cell cycle is often dysregulated in diseases such as cancer and neurodegeneration, and CRL components are often mutated or dysregulated in many of these diseases. While the neddylation inhibitors pevonedistat and TAS4464 are currently being tested in the clinic, these drugs affect neddylation nonspecifically and may disturb many pathways not related to the cell cycle. In this regard, the next generation of drugs will aim to target individual CRLs or specific interactions between CRL components or CRLs and their substrates. Promising examples for this approach include substrate-specific small-molecule enhancers (e.g., lenalidomide, pomalidomide, and thalidomide) that promote specific ubiquitination of CRL4 substrates (e.g., Ikaros, Aiolos, Casein kinase 1a, and Cereblon)^104,105^. As proper cell proliferation requires the timely recycling or degradation of ~100,000 different proteins per cell, it is reasonable that approaches utilizing small molecules tailored to the modulation of distinct substrate receptors will open new possibilities for specific targeted therapies.

A new prospect for drug development may be to better understand how CRLs are recruited to specific cell components. CRL4 was recently shown to be loaded onto chromatin by the structural DCAF RepID, the recruitment of which is crucial for regulating several aspects of cell cycle progression. Other CRL families may also be recruited to specific cellular locations using a similar principle. This newly discovered mechanism of action necessary for CRL activity may be, in turn, targeted by small-molecule inhibitors (i.e., interaction between the “recruiter” RepID and CUL4). An example of the value for this new strategy is shown by the cellular pathways leading to CDT1 destruction. Modulation of CRL4 recruitment to chromatin in RepID-deficient cells showed a synergistic effect with the inhibition of SKP2 by stalling CDT1 degradation and leading to accrued cell death. Thus, future combinatory approaches targeting a “recruiter” from one CRL together with the targeting of a substrate receptor from a different CRL may boost therapeutic success since a cell may use redundant CRL pathways to destroy the same protein.
